# Supplementary material for: Application of Augmented Reality Technology as a Dietary Monitoring and Control Measure Among Adults: A Systematic Review
Source: Nutrients. 2025 Dec 12;17(24):3893. doi: 10.3390/nu17243893 (PMC12735909; doi:10.3390/nu17243893)
Supplement: Supplementary file 1 [file nutrients-17-03893-s001.zip › nutrients-3990699-supplementary.pdf]

Supplementary Table S1.

Search period: January 2000–May 2025
Search type: Systematic search

Database	Search Strategy	Search Limits
**PubMed**	(“Augmented Reality” [Mesh]<br>OR "augmented reality"<br>OR "mixed reality"<br>OR "extended reality"<br>OR "AR technology")<br><br>AND<br>("Dietary Records"[Mesh]<br>OR "Nutrition Assessment"[Mesh]<br>OR "dietary assessment"<br>OR "dietary control"<br>OR "dietary monitoring"<br>OR "dietary behavior"<br>OR "food intake"<br>OR "portion size")	• Publication date: 2000-2025<br>• Humans<br>• English<br>• Adults (18+)<br>• Peer-reviewed or academic journals<br>• Full Text
**Embase**	('augmented reality'/<br>OR 'augmented reality'<br>OR 'mixed reality'<br>OR 'extended reality'<br>AND<br>('dietary intake'/<br>OR 'nutrition assessment'<br>OR 'dietary behavior'<br>OR 'dietary assessment'<br>OR 'dietary control'<br>OR 'dietary monitoring'<br>OR 'portion size'<br>OR 'food intake')	• Publication date: 2000-2025<br>• Humans<br>• English<br>• Adults (18+)<br>• Peer-reviewed or academic journals<br>• Full Text
**CINAH**	("Augmented Reality"<br>OR "augmented reality"<br>OR "mixed reality"<br>OR "extended reality")<br>AND<br>("Nutrition Assessment"<br>OR MH "Dietary Intake"<br>OR "dietary assessment"<br>OR "dietary control"<br>OR "dietary monitoring"<br>OR "dietary behavior"<br>OR "food intake"<br>OR "portion size")	• Publication date: 2000-2025<br>• Humans<br>• English<br>• Adults (18+)<br>• Peer-reviewed or academic journals<br>• Full Text

Supplementary Table S2.

Study	Instrument	Description	Scoring Range	Mean (SD) if Reported
Saha et al. (2022)	CSUQ (Computer System Usability Questionnaire)	Standardized 19-item scale assessing usefulness, information quality, and interface quality	1-7 (lower scores = higher satisfaction)	Mean: NR; Satisfaction; 73% strongly agreed the app increased productivity
Saha et al. (2022)	User Satisfaction Survey	Custom survey evaluating satisfaction and ease of use	1-5 Likert Scale	80% satisfied/extremely satisfied
Brown et al. (2019)	Custom Usability Questionnaire	11 item tool assessing ease of aligning device, clarity, and usefulness	1-5	72.5% reported tool easy to use

Rollo et al (2017)	Custom Likert-based Usability survey	Measures ease of use, usefulness, clarity, and alignment	1-7	73.3% agreed/strongly agreed tool aided appropriate serving
Lam et al. (2021)	System Usability Scale	Measures perceived usability of a product/system	5-point Likert Scale	37.5% accuracy improvement
Alturki & Gay (2019)	Qualitative Acceptability Assessment	Interviews and observations assess ease of use and interactivity	N/A	100% found the app easy to navigate and use
ChanLin et al. (2019)	Embedded Evaluation Metrics	Post-test satisfaction and engagement	1-5	100% improved dietary awareness
Stutz et al. (2014)	Acceptability Survey	Acceptability of mobile AR vs. other methods	N/A	Qualitative acceptability themes

Abbreviations: N/A = Not Available; SUS = System Usability Scale; CSUQ = Computer System Usability Questionnaire
